# Real-Life Application of a Wearable Device towards Injury Prevention in Tennis: A Single-Case Study

**DOI:** 10.3390/s22124436

**Published:** 2022-06-11

**Authors:** Iztok Kramberger, Aleš Filipčič, Aleš Germič, Marko Kos

**Affiliations:** 1 Faculty of Electrical Engineering and Computer Science, University of Maribor, Koroška Cesta 046, 2000 Maribor, Slovenia; marko.kos@um.si; 2 Faculty of Sport, University of Ljubljana, Gortanova 22, 1000 Ljubljana, Slovenia; ales.filipcic@fsp.uni-lj.si (A.F.); ag6608@student.uni-lj.si (A.G.)

**Keywords:** tennis, training, data-based coaching, shot recognition, wearable device, workload, recovery

## Abstract

The purpose of this article is to present the use of a previously validated wearable sensor device, Armbeep, in a real-life application, to enhance a tennis player’s training by monitoring and analysis of the time, physiological, movement, and tennis-specific workload and recovery indicators, based on fused sensor data acquired by the wearable sensor—a miniature wearable sensor device, designed to be worn on a wrist, that can detect and record movement and biometric information, where the basic signal processing is performed directly on the device, while the more complex signal analysis is performed in the cloud. The inertial measurements and pulse-rate detection of the wearable device were validated previously, showing acceptability for monitoring workload and recovery during tennis practice and matches. This study is one of the first attempts to monitor the daily workload and recovery of tennis players under real conditions. Based on these data, we can instruct the coach and the player to adjust the daily workload. This optimizes the level of an athlete’s training load, increases the effectiveness of training, enables an individual approach, and reduces the possibility of overuse or injuries. This study is a practical example of the use of modern technology in the return of injured athletes to normal training and competition. This information will help tennis coaches and players to objectify their workloads during training and competitions, as this is usually only an intuitive assessment.

## 1. Introduction

The role of data in sports has increased significantly in recent years. The data-driven approach is the subject of scientific research and constant development. Professional, junior, and recreational athletes collect, monitor, and analyze data to gain information about their performance or the possibility of improvement [1]. With the increasing availability of data in tennis tournaments, it is possible to study player performance from tactical, technical, mental, and physical perspectives [2].

Most commercially available micro-engineered devices contain microsensors such as accelerometers, gyroscopes, magnetometers, and global positioning systems (GPS). Some available inertial measurement units (IMUs), such as microelectromechanical sensors (MEMS), contain one or a combination of these sensors to capture and analyze the movements of athletes during many sport activities [3]. Because of the IMU’s small size and minimum weight, it is especially appropriate for swing-based sports such as racket sports, volleyball, or golf, where any additional weight on the arms would most likely disturb the player and have some influence on the player’s performance [4]. This gives rise to the potential for athletes to be observed outside of a laboratory setting and in their natural training or competitive environment [5].

These devices, commonly referred to as wearable sensors, provide detailed real-time motion analysis of athletes during competition and training and offer an alternative to labor-intensive video coding and testing in a laboratory setting [3,5]. The wearable device is worn on the wrist or chest and can collect data on the athlete’s kinematics, heart rate, workload, skin temperature, and sleep patterns. It can also monitor movements of the body or body segments [3,5,6], distinguish between amateur and recreational tennis players [7], and recognize tennis stroke types [8] using an inertial measurement unit.

Wearable sensor systems are an emerging tool for assessing athletic activity and can be used to quantify the athlete’s external workload in racket sports [9,10,11,12]. Training load can be described as external and/or internal, depending on whether we are referring to measurable aspects that occur internally or externally to the athlete. The organization, quality, and quantity of exercise determine the external load, which is defined as the physical work prescribed in the training plan [13]. In tennis, wearable devices attempt to quantify or estimate gross motion to provide a measure of the external load. The relevance of these metrics to training specifications is twofold. First, athletes must be exposed to sufficient load to achieve the desired performance improvements. Second, the training load (acute or chronic) must not be so high as to increase the risk of injury. In this pursuit of optimal training load, monitoring tools are critical [10].

The use of wearable devices in tennis has expanded over the past decade in research, testing and screening, and daily monitoring of tennis players. Genevois et al. [14] used relatively simple tools to provide coaches with practical information to calculate and optimize training load, paying particular attention to the rate of the perceived exertion (RPE) method for a session, calculation of the monotonicity index, and calculation of the acute/chronic workload ratio. The RPE scale measures the perceived intensity level of a physical activity. 

Giménez-Egido et al. [11] used wearable devices to assess the optimization of teaching and learning using a comprehensive approach. Based on the data collected, coaches were able to focus on quantity, variability, and creativity in training, assessing health parameters, improving cognitive motivation, and analyzing individual technical-tactical patterns to correct errors or training deficits.

Filipcic et al. [15] conducted a single-case study using a wearable device that provided good insight into the entire training and competition process and could serve as a basis for determining workload indicators for female professional tennis players.

Modern tennis players are faced with crowded schedules that force them to use different recovery strategies. Therefore, recovery must be fine-tuned with accurate quantification of its effects, especially with respect to training-induced fatigue. A periodized approach to recovery is a first step toward interventions based on the interactions between training load, training content, and perceived recovery [16]. Heart rate (HR), recovery rate (HRR), and heart rate variability (HRV) after exercise are commonly used to measure the cardiovascular parasympathetic function noninvasively [17] and the influence of training status, different types of training, gender, and age on heart rate variability (HRV) indices in athletes. The predictability of HRV is also considered in overtraining, athletic status, and athletic performance. The cardiac autonomic imbalance observed in overtrained athletes implies changes in HRV and, therefore, suggests that heart rate variability may provide useful parameters for detecting overtraining in athletes [18].

The aim of this study is to develop a model for data-driven monitoring of specific tennis loads during training and matches using a personal wearable tracker and mobile app. Targeting specific tennis loads will also allow monitoring of the athlete’s response to training load and the quality of the recovery process using morning HRV measurements. Using numerous performance indicators and features, coaches and players can monitor, analyze, and plan the workload, content, and efficiency of tennis practice and matches. The analysis is carried out in real time on a daily basis and is based on objective and measurable data. The standard values were determined and set based on scientific studies that analyzed workload during official tennis matches and tennis training and on the practical experience of tennis coaches.

## 2. Materials and Methods

A professional female tennis player (age: 21 years; height: 167 cm; weight: 64.5 kg; years played: 16; WTA ranking position at the time of the study: 505) agreed to be observed for six months. At the start of observation, she had no severe muscle, joint, or bone injuries. The athlete was informed of the benefits and risks of the study before signing an institutionally approved informed consent form to participate in the study.

The tennis player did not practice or compete for eight months due to lower back pain. The results of the injury examination (MRI, ultrasound, physiotherapy tests) did not reveal the true cause of the pain. Therefore, we decided to monitor the workload with a personal wearable tracker and a mobile app (the Tennis Armbeep System—TAS) to prevent a possible overload and, thus, a recurrence of the pain. The process of monitoring, analysis, and planning was based on the athlete’s individual daily measurements during the preparation, pre-competition, and competition phases of an annual season.

The miniature wearable device (Armbeep; version 2.0, Biometrika, Maribor, Slovenia) was designed with low weight and small form in mind. The device is worn on the player’s dominant arm, above the ulnar head. The device measures 39 mm × 33 mm × 12 mm and weighs only 12 g and, therefore, does not influence the player’s feeling for the racket and/or the player’s performance. Because it is lightweight, it can also detect vibrations from the racket very efficiently. The vibrations are a good indicator of whether a player is hitting the ball with the racket in the sweet spot (the optimal spot on the racket head). The housing is made from plastic material, which is resistant to moisture and sweat. The housing is also waterproof, so that the printed circuit board (PCB) and the sensors inside do not become contaminated and oxidized. This is especially important for battery contacts. The device can also be placed and worn under a sweatband.

**Figure 1:**
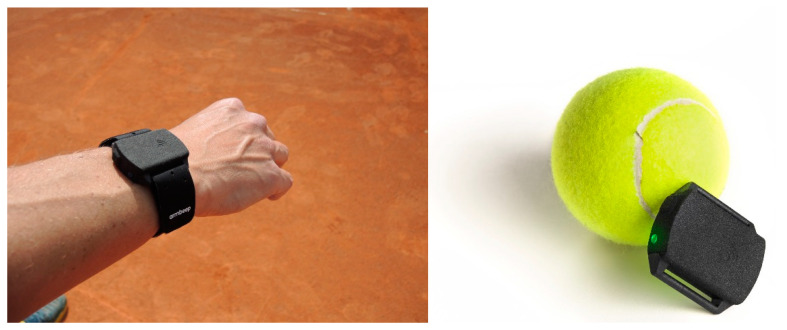
Armbeep device placement and size comparison.

The wearable device can monitor and record movement and biometric information of a player during a sports activity. The device was recently upgraded to version 2.0, where the pulse sensing circuit and IMU were replaced, and its connectivity was also upgraded. The main communication channel is now via Bluetooth, which enables the device to connect to smart mobile devices (phones, tablets) wirelessly. The IMU was replaced with a unit with a larger detection range. The device also grew minimally in size and weight. A graphical representation and size comparison are depicted in Figure 1. The device is placed laterally on the ventral surface of the forearm on the dominant arm. This position is favorable for effective arm movement detection, racket vibration monitoring, and also for reliable pulse and blood oxidation measurements. The device incorporates a 6-DOF MEMS IMU (DOF—degrees of freedom, MEMS—microelectromechanical system) for monitoring arm accelerations and movement. With its selectable accelerometer range of up to ±30 g, it is suitable for high-impact sport applications. Such a high accelerometer range is needed for accurate tennis stroke monitoring; otherwise, some clipping can occur. The gyroscope supports angular velocity measurements up to ±4000 dps. The initial sensitivity of both the accelerometer and gyro is factory-calibrated, so no additional calibration is needed. The MEMS IMU offers communication over a 7 MHz SPI bus, which is useful for reading the internal 4 kB FIFO buffer in burst modes. The IMU also has a programmable data rate. Rates are supported from 4 Hz up to 4.5 kHz for the accelerometer and 9 kHz for the gyro. We used a data rate of 1.25 kHz, because we are convinced that this is enough for detecting and monitoring racket vibrations and string oscillations. Previous work also confirms this [19]. Besides the movement, the device is also able to monitor biometric information during sports activity. The device is equipped with a circuit for pulse rate measurement using the reflective photoplethysmography (PPG) method. The device supports measurement with two different light sources (green and yellow) for more robust pulse detection and estimation. The pulse rate (PR) value and pulse rate variability (PRV) are measured and stored during minimum or no wrist movement. The moments when the player’s hand is not moving are detected with the IMU. More reliable readings are obtained in this way. The device is powered with a 155 mAh lithium-polymer (LiPo) battery with a nominal voltage of 3.7 V. With one charge, the device can operate for around 6 h in active mode, which is enough for typical tennis training sessions and matches. 

With the Armbeep wearable device, data were collected throughout the session. The IMU collected the accelerometer and gyroscope data for each stroke, and the PPG circuit collected the HR and HRV. Acceleration values were measured in all three axes, and only movements with amplitude values greater than 1 G at the point of impact were detected as a stroke. Hadžić et al. [9] reported that, based on the results, the Armbeep sensor can be considered a valid and reliable tool for measuring the actual number of strokes and monitoring hitting load during tennis practice and matches.

### 2.1. Data Acquisition and Processing

**Figure 2:**
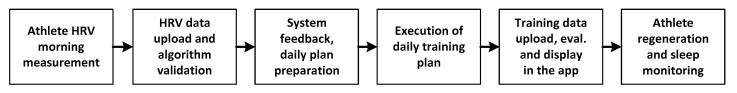
Display of the athlete’s daily activities and use of the smart wearable sensor device.

During the six-month training and competition period, the athlete recorded all tennis practice and matches in the singles and doubles categories as well as the morning HRV measurements. The athlete performed a 3–5 min HRV measurement each morning before any other activity. She transferred the data to her personal profile via the mobile app. Based on the HRV data evaluated with the scoring algorithm, the system assesses the level of fatigue, with three values displayed on the calendar (green, yellow, orange). Based on the displayed values in the TAS, the coach and the athlete perform the planned daily training schedule. The athlete also measured all planned daily tennis activities with a monitoring device on her wrist. After each training session, she transferred the collected data via the mobile app to her personal profile in the TAS. The process is depicted in Figure 2. 

For the study purposes, the data in the athlete’s personal profile were exported in CSV format for further processing.

The data were collected over a period of six months (from March until November). In this time interval, the observed athlete went through three training periods: preparation, pre-competition, and competition. In the preparation period, the athlete had 47 training sessions and five matches and was able to carry out 50 HRV measurements. In the pre-competition period, the athlete had 34 training sessions and nine matches and completed 43 HRV measurements. In the competition period, the athlete had 58 training sessions and 35 tennis matches and was able to complete 66 HRV measurements. As mentioned previously, the HRV measurements were performed in the morning before any activity. 

### 2.2. Variables

The indicators used in this study, recorded by a smart wearable sensor device, were divided into four groups: session time characteristics, physiological and movement indicators, and tennis-specific performance indicators. These were divided into two subgroups: shot and rally indicators. All of the variables, with descriptions, units, and the descriptive statistics’ parameters, are listed in Table 1.

Basic information about the current activity is stored for each session during practice or a match. A unique session ID is generated, and the unique athlete ID is also stored in the header of the session file along with the session date and time. The time and date of when the session is eventually uploaded to the cloud service are stored separately.

In Table 1, the mean values for information and performance indicators are presented, and standard deviation (SD) is also presented, for better statistical insight (data scatter). The first group of performance indicators is linked to average session duration, which includes active and inactive moments of activity. Active time is defined as the sum of all rally times in a practice session or match. A rally is when the athlete is exchanging shots with the opponent, and it can be tracked with a wearable device as moments when tennis shots are detected with a minimum pause (7 s). A new rally starts when the time between two consecutive shots is longer than 7 s. The percentage of active time, average rally time, and average rest time are also monitored.

The next group of performance indicators is heart rate (HR)-related. The average, minimum, and maximum heart rates are monitored (AvgHR, MinHR, MaxHR), whereas the percentages are also recorded in the high-, moderate-, and low-HR zones. HighHR is defined as the percentage of a session time when the HR was in the 86–100% zone of maximum individual HR. ModerateHR is defined as the percentage of a session time when the HR was between 71% and 85% of the maximum individual HR, and LowHR is defined as the percentage of a session time when the HR was in the 50–70% zone of the maximum individual HR. Recovery performance indicators also fit in the HR performance indicator group. Performance indicators Recovery20Count, Recovery60Count, and Recovery120Count are defined as the percentage in HR decrease in a corresponding time window (20, 60, and 120 s, respectively). Recovery 20 BPM, recovery 60 BPM, and recovery 120 BPM are defined as the number of HR BPM (BPM—beats per minute) drops where the athlete’s HR was in the corresponding time window after a HR drop from a high value.

The next group of performance indicators is cardio load indicators. The first one is the CardioLoad index, which is calculated for the last seven days of activity with the following formula:

(1) $$CL=\frac{sum\;of\;last\;week\;sessions}{sum\;average\;of\;last\;four\;weeks}$$

where *CL* stands for CardioLoad, and the rest are the last seven days’ sum and 28 days’ session sum divided by 4 (one week average). The individual cardio load index is determined based on %*VO*_2_ (body oxygen consumption) measurements, supported by the new Armbeep 2.0 device. The equation is:

(2) $$\%VO_{2}=\frac{VO_{2}}{VO_{2max}}=\frac{0.002{\left(current\;HR\right)}^{2}-0.13\left(current\;HR\right)+2.3}{0.002{\left(max\;HR\right)}^{2}-0.13\left(\;max\;HR\right)+2.3}$$

where *current HR* is the *HR* in a given moment, and the *max HR* is the maximum *HR* in the current session. The partial in-session cardio load index is then determined from the table values (cardio load EPOC). The values are presented in Table 2. For individual sessions, the partial cardio load index values are summed up. An individual session is divided into five time zones (0–5 min, 5–10 min, 10–30 min, etc.). For a weekly cardio load estimation, individual sessions’ cardio load values are summed up for a one-week period. The optimum value of a CL index is between −25% decrease and +25% increase compared to a four-week average value. Values that are out of this bound can lead to an increase in risk of overuse types of injuries and, worse, gain of endurance types, due to a lack of regeneration [20].

The next type of performance indicator is movement. These performance indicators are calculated once per second according to the Valencell propriety algorithms embedded in their module, which is a part of the Armbeep 2.0’s hardware (Biometrika d.o.o, Maribor, Slovenia) and is responsible for movement tracking. Different movements around the tennis court are tracked and classified, such as running, sprinting, walking, and standing (no or little movement). Movement is detected and labeled every second. Values are presented in percentages.

The following are the shot type performance indicators. Shots such as forehand, backhand, and overhead type shots (serve, smash) are detected and classified. Performance indicator “shots” is the sum of all shots in one session, and the average for a timespan of six months is presented in Table 1 (like all other indicators). Shots labeled as “ShotsOther” are all of the detected shots that are not classified as any other shot type.

The next type of indicator is in close relation to the previous ones, and is also about shots, but more the frequency of shots than the type. Performance indicators are calculated such as shots per hour and shots per rally. The minimum and maximum number of shots per rally are also monitored, and the total number of rallies is also determined. The number of shots per rally is presented in three categories: low, moderate, and high. All are presented in percentages. 

The next category is the tempo of shots, where six different performance indicators are monitored. Tempo is presented as the number of shots per minute (frequency of shots per minute), whereas TempoMin and TempoMax are indicators for the minimum and maximum number of tempos per session. TempoLow is defined as the percentage of shots per minute in the range of 1 to 10; TempoModerate is defined as the percentage of shots per minute in the range of 11 to 19; and TempoHigh is defined as the percentage of shots per minute exceeding 20 shots per minute. 

The last presented category of performance indicator regards shot power and hitting load. The ShotsPower indicator is the value of the average power of shots measured on the wrist in g. The sum of all shots’ accelerations is divided by the number of shots. The average is calculated for each individual session. The power of shots is then also monitored in three ranges. The ShotsPowerLow is defined as the percentage of shots in the range of 1 to 10 g; ShotsPowerModerate is defined as the percentage of shots in the range of 11 to 19 g; and ShotsPowerHigh is defined as the percentage of shots with wrist acceleration over 20 g. The HittingLoad (HL) performance indicator is, in a sense, an armload index, related to the number and power of shots in one hour of play. It is calculated according to the equation:

(3) $$HL=\frac{\sum SP*ATh*\left[\left(\frac{AT}{ST}\right)*\left(\frac{AT}{3600}\right)\right]}{10}$$

where *SP* stands for the sum of power for all shots in one hour; *ATh* stands for active time per hour; *AT* stands for active time; and *ST* stands for session time. The *HL* values in Table 1 show that the hitting load is higher for practice sessions than for match sessions. This is due to the fact that the duration of training sessions is longer, and, therefore, the values for hitting load are higher [10].

## 3. Results

In Table 1, the descriptive statistics were reported as mean ± standard deviation for all numerical variables, along with the names, descriptions, and units. The table includes all variables that we used to monitor and determine the daily, weekly, or monthly workload of athletes for training and competition.

Time variables (SessionTime, ActiveTime, ActiveTimePercentage, AvgRallyTime) are expected to be higher during training; this is consistent with the basic principles of sports training, which recommend exceeding (overreaching) the competitive load during training [21]. The physiological indicators expressed by different heart rate values (AvgRestTime, AvgHR, MinHR, MaxHR, HighHR, ModerateHR) are higher during matches. Tennis players are exposed to both physiological (movement and execution of tennis shots) and psychological stress during the match. As Smekal et al. [22] note, one of the most important factors affecting the heart rate indicators is the length of the rallies (AvgRallyTime).

The indicators that determine the decrease in maximum heart rate after physical activity (TotalRecoveries, Recovery20Count, Recovery60Count, Recovery20BPM, Recovery60BPM) are higher during games, because the timing and duration of rallies and rest periods are determined by the rules. Therefore, we did not record any event during the matches and training sessions that would determine the drop-in heart rate after 120 s of rest (Recovery120BPM, Recovery120Count).

Cardio load (CardioLoad) was calculated based on a developed algorithm and determines the weekly cardio load of athletes based on the total physiological load. Cardio load depends on the distribution of recovery and rest periods and the content, intensity, frequency, and volume of daily training when considering the total weekly cardio load. Due to the aforementioned influence of psychological stress and high-intensity and long-duration activities during competitions, the cardio load is higher during matches.

With TAS, we were (for the first time) able to collect data on the athlete’s movement (movement, sprinting, running, walking, standing) during training and competition. There are no differences between matches and training in terms of the total movement index or the percentage of the player’s movement in other speed classes.

During an annual training season, we collected data on the average number of shots per session and shots per hour that had higher values during practice (Shots, ShotsPerHour). Based on our shot detection and recognition algorithm, we categorized shots into four groups: overheads, forehands, backhands, and other shots. It was expected that both the total number and percentage of baseline shots would be higher during the practice (ShotsForehand, PForehand, ShotsBackhand, PBackhand, ShotsOther), while the values of serves (ShotsOverhead, POverhead) would be higher during matches. The conclusion is consistent with the findings that coaches pay too little attention to the serve and the routine of the serve [23].

Just as the duration of rallies, the number of shots (ShotsPerRally) is higher in practice sessions. Since rallies in a match always start with a serve or return of serve, this has a significant impact on the higher intensity of the movements and, thus, on the shortening of the duration. Researchers [24,25] found that up to 70% of rallies in matches end within four shots of both players. Other performance indicators (ShotsPerRallyLow, ShotsPerRallyModerate, ShotsPerRallyHigh, ShotsPerRallyMin, ShotsPerRallyMax) that determine the number of shots in rallies indicate higher values during training, resulting from training sessions or parts of training sessions that target specific warm-up exercises or repetitive game situations. There are no significant differences in the number of rallies (RalliesTotal) between the two types of session observed.

An important group of performance indicators measures the number of shots per minute (Tempo, TempoLow, TempoModerate, TempoHigh, TempoMin, TempoMax). This means that the number of shots per minute determines the expected number of shots in most rallies (especially those shorter than 1 min). Since rallies in the game start with the serve and continue with the return of serve, the values for shots per minute (tempo or rally speed) are higher in matches than in practice. In practice, rallies begin with the baseline shots, serve, or return of serve. For this reason, the values for the shots per minute are lower in practice. Tempo is an important factor in tennis, because it includes cognition, perception, anticipation, reaction, accuracy, speed of movement, agility, and the technical competence of players [26]. In our opinion, all of these can also have a decisive influence on the outcome of a match, namely, the ability to play at a higher tempo than an opponent.

The power of the shot is measured in a 300 ms time window in the zone of the tennis shot. The IMU contains data 150 ms before and 150 ms after the point of impact. The different values of the impact power (ShotsPower, ShotsPowerLow, ShotsPowerModerate, ShotsPowerHigh) determine the ability to accelerate the racket through the impact zone directly and the technical efficiency of the shot indirectly, both from a motor and biomechanical point of view [9,10,11,27].

Hitting load (HittingLoad, HL) is calculated based on an algorithm and determines the total load on the dominant limb. Hitting load indicates the number and power of shots in one hour of play. Individual analysis of a single tennis player allows monitoring of hitting load during practice and matches. Large deviations from the daily hitting load can increase the risk of injury [15].

Based on the performance indicators presented in Table 3, we obtained feedback on the efficiency of regeneration and the current state of the athlete based on the athlete’s morning measurements. Based on the HRV index value and other related indicators, we derived or adjusted the planned daily training load.

The variables in Table 3 are common HR and HRV variables (MinBPM, MaxBPM, AvgBPM, SDNN, RMSSD, pNN50, HRVScore), except the *hrv* index, which is calculated according to the procedure below. First, we calculate an intermediate value *hrv′* by the following formula:

(4) $$hrv^{\prime }=\frac{SDNN}{\max SDNN}+\frac{RMSSD}{\max RMSSD}+\frac{pNN50}{\max pNN50}+\frac{HRVScore}{\max HRVScore}$$

where *SDNN* stands for standard deviation of NN (R-R) intervals; max *SDNN* stands for absolute maximum deviation of *SDNN*; *RMSSD* stands for the root mean square of successive heartbeat interval differences; max *RMSSD* stands for the absolute maximum deviation of *RMSSD*; *pNN50* stands for a proportion of the number of successive NN intervals that differ by more than 50 ms; max *pNN50* stands for the absolute maximum deviation of *pNN50*; *HRVScore* is the variation of the time between heartbeats; and the max *HRVScore* is the absolute maximum deviation of the *HRVScore*. The next step is to determine the absolute maximum deviation of *hrv′*:

(5) $$maxdev\_hrv^{\prime }=MAX\left(ABS\left(hrv^{\prime }\_dev;\left[1:n\right]\right)\right)$$

where *hrv′_dev* is the deviation of *hrv′*, and n is the consecutive *hrv′* value measurement index. The final value of the *hrv* index is calculated according to the following formula:

(6) $$hrv=10*\frac{hrv^{\prime }}{maxdev\_hrv^{\prime }}$$

where *hrv′* is the intermediate value of HRV, and *maxdev_hrv′* is the absolute maximum deviation of *hrv′*. Formula (6) gives us a range of values for *hrv* from −10 to 10. Values from −7 to 8 coincide with low risk of injury, whereas other values indicate a greater possibility of injuries [28].

**Figure 3:**
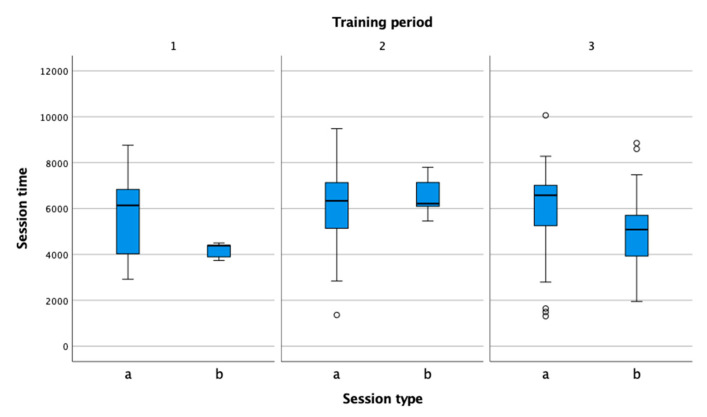
Comparison of practice (a) and match (b) session time in the preparation (1), pre-competition (2), and competition periods (3).

In the figures, performance indicators were compared by session type (practice, match) and by training period (preparation, pre-competition, competition). Information in the figures is presented in box-plot form. Circles and asterisks represent outliers. In all three observed training periods (Figure 3), the session duration was higher in training than in matches. The duration of matches was shortest in the preparation phase, which is consistent with the theoretical recommendations that athletes should pay the most attention to preparation rather than matches in this phase [29]. In the pre-competition phase, the number of matches increased, including unofficial sparring matches, and corresponded to the number of training sessions. In the competition period, the volume of training sessions remained at the same level compared to the previous period, while the volume of competitions decreased.

**Figure 4:**
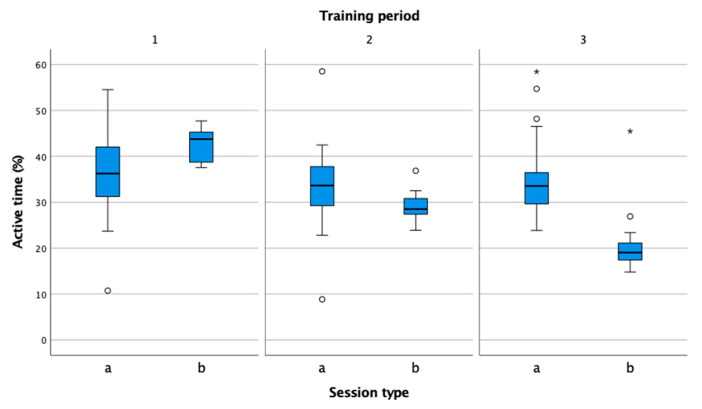
Comparison of percentage of active time in practice (a) and match (b) in the preparation (1), pre-competition (2), and competition periods (3).

In Figure 4, the percentage of active time compared to total training time does not differ in the three periods, which is consistent with the values recommended by experts for female tennis players [15]. In the competitions, the values of the performance indicators from the period decreased, and in the competition period, they correspond to the values measured in many studies [30,31,32,33].

**Figure 5:**
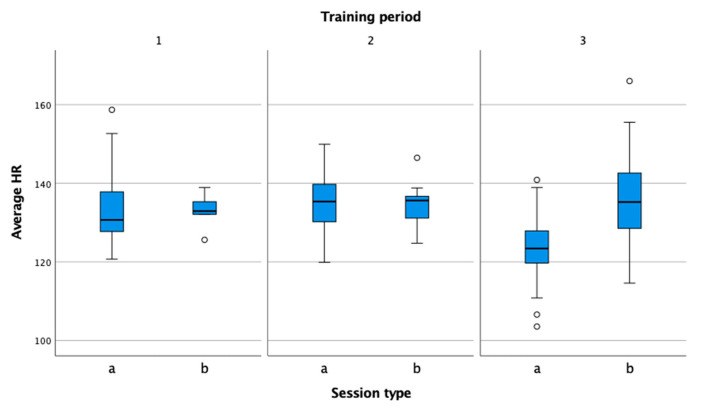
Comparison of average heart rate in practice (a) and match (b) in the preparation (1), pre-competition (2), and competition periods (3).

The difference in average heart rate (Figure 5) between training and competition was greatest during the competitive phase. During this period, the athlete completed the most official matches. According to the findings of Mendez-Villanueva et al. [34] and Smekal et al. [22], the high intensity of the play and the mental factors increase the level of physical and physiological demands.

**Figure 6:**
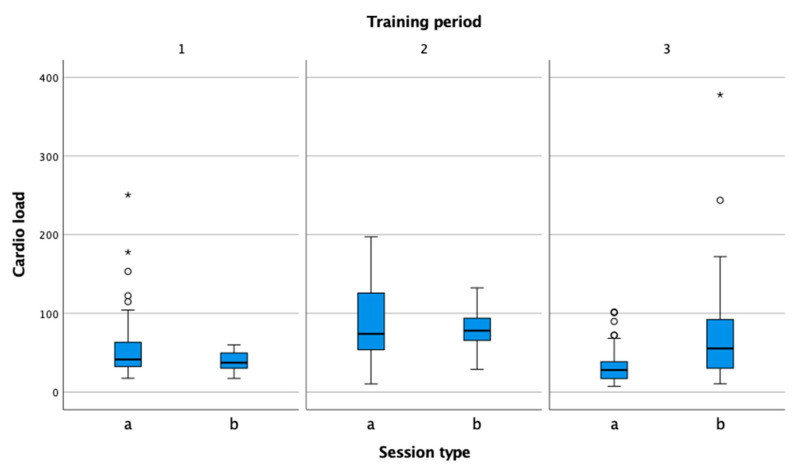
Comparison of practice (a) and match (b) cardio load in the preparation (1), pre-competition (2), and competition periods (3).

The values for cardio load (Figure 6) show that the load increases gradually from the preparation phase to the pre-competition phase, both in the practice sessions and in the competitions. In the competition phase, the physiological load is reduced in training so that the tennis player can regenerate properly and prepare for the match optimally.

**Figure 7:**
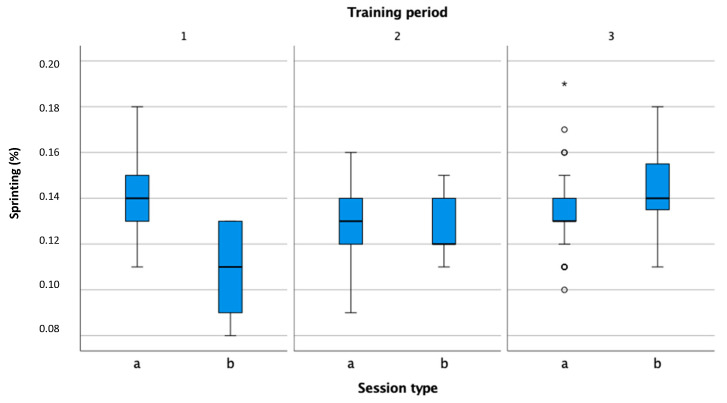
Comparison of the percentage of time in the sprinting speed zone; percentage in practice (a) and match (b) in the preparation (1), pre-competition (2), and competition periods (3).

The speed of movements in practice and in a match has not been measured in previous studies. Values that measure the percentage of movements accurately in four speed zones provide information about the demands that occur during a match. Figure 7 shows the percentage of time in the sprinting zone, in which the athlete was moving at a speed greater than 16 km/h. Tennis coaches can determine the movement load more accurately during practice using reference values. The analysis of the speed of movement has a significant impact on the determination of the movement loads of athletes and, consequently, with appropriate dosage, on the reduction in injuries of the lower extremities.

**Figure 8:**
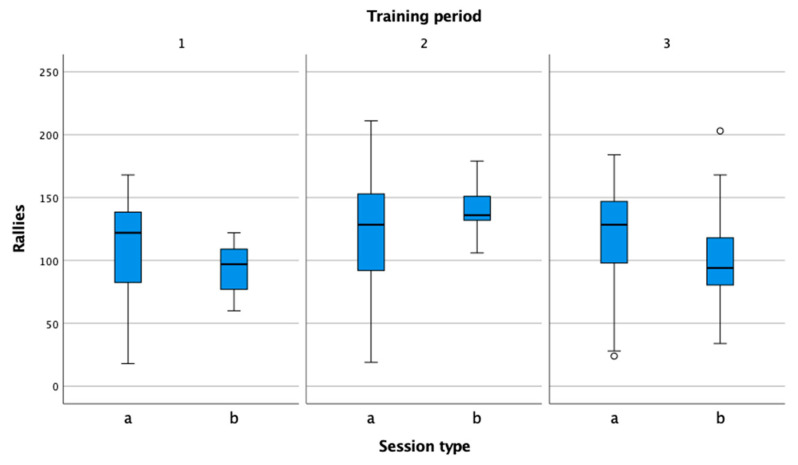
Comparison of the number of rallies in practice (a) and match (b) in the preparation (1), pre-competition (2), and competition periods (3).

The number of rallies in a session (Figure 8) helps tennis, fitness, and conditioning coaches to determine the number of repetitions of individual exercises objectively and, thus, to control the workload systematically during the preparation period.

**Figure 9:**
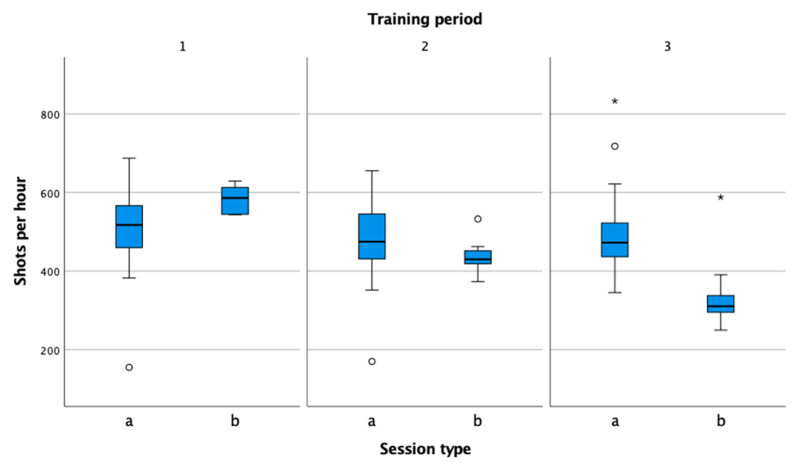
Comparison of shots per hour in practice (a) and match (b) in the preparation (1), pre-competition (2), and competition periods (3).

Shots per hour (Figure 9) is a relative variable that allows you to compare specific tennis loads at different stages of training and competition. In training, the values are in line with the studies conducted so far and range between 400 and 600 shots [15,35,36]. The number of shots per hour during matches is usually lower (pre-competition, competition). Important factors influencing the number of shots per hour in competition are the surface, the playing style of the player and the opponent, and the level of play. 

**Figure 10:**
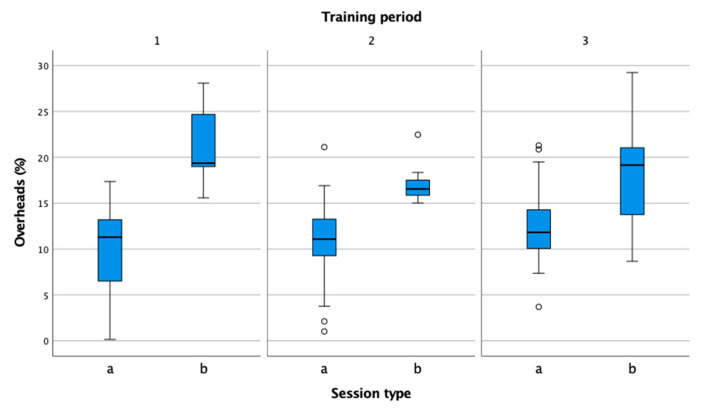
Comparison of overhead percentage in practice (a) and match (b) in the preparation (1), pre-competition (2), and competition periods (3).

The percentage of serves (Figure 10) is 20% or more in official matches [37,38]. In training, it is important to be as close as possible to the competition level, so a high percentage of rallies begin with a serve. Although we spent half of our training time serving and continuing the rally, we did not come close to the competition values. It is important for tennis coaches to create match-like conditions in practice.

**Figure 11:**
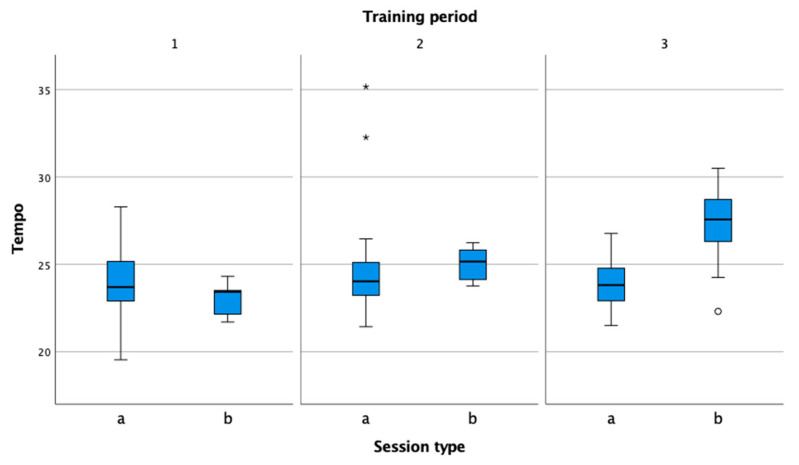
Comparison of tempo in practice (a) and match (b) in the preparation (1), pre-competition (2), and competition periods (3).

**Figure 12:**
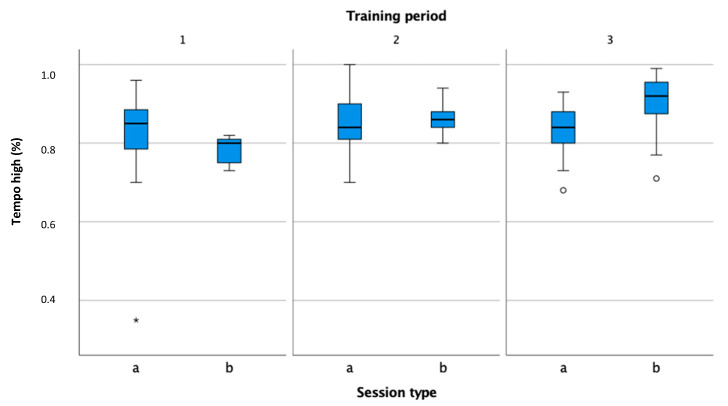
Comparison of high tempo percentage in practice (a) and match (b) in the preparation (1), pre-competition (2), and competition periods (3).

With the exception of the preparation phase, the tempo (Figure 11) is higher during matches. All rallies in matches start with a serve, which tennis players execute faster than other shots, which, in turn, leads to a higher number of shots per minute [2,36]. This also affects the percentage of rallies where the tempo is higher than 21 shots per minute (Figure 12).

**Figure 13:**
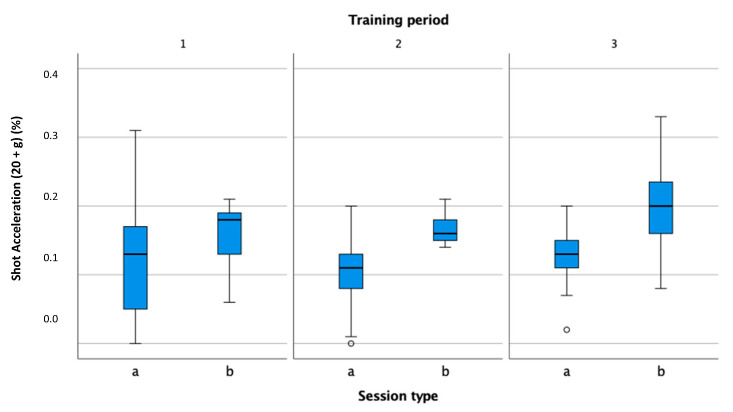
Comparison of high shot acceleration (20 g or more) percentage in practice (a) and match (b) in the preparation (1), pre-competition (2), and competition periods (3).

In all of the observed periods, the percentage of shots with high force (more than 20 g) was higher during matches, which is due to the structure of training, where a certain part also consists of warm-up, cool-down, and technical exercises and where tennis players do not perform kicks with high speed (Figure 13).

**Figure 14:**
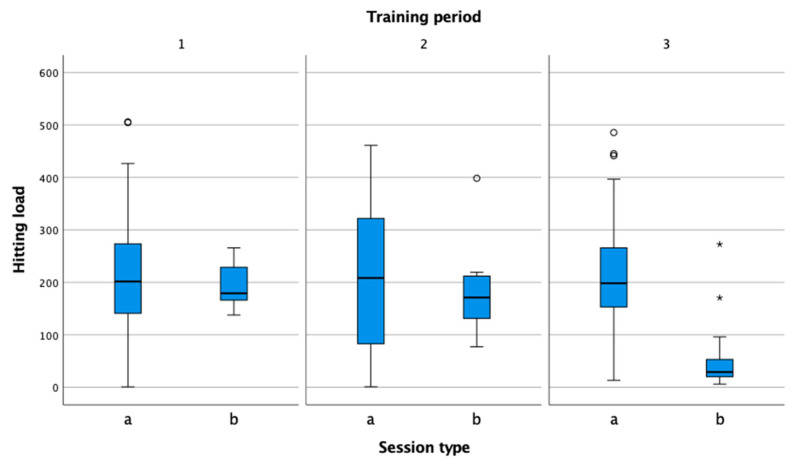
Comparison of hitting load in practice (a) and match (b) in the preparation (1), pre-competition (2), and competition periods (3).

Hitting load (Figure 14) is reduced significantly only during the competitive period, when the tennis player participates only in official matches limited by the rules of tennis. While in the preparation and pre-competition periods, the duration of matches according to the training schedule was also longer, and, therefore, the values for hitting load were higher.

### 3.1. Practical Use

The Tennis Armbeep System allows the collection of data that can be exported through the online platform for later statistical analysis. The other part of the system is the mobile app. The mobile app is a user interface designed to display data to users (athletes, coaches, parents). Each user has their own profile, with personal information that is important for data analysis (age, gender, body weight and height, dominant limb, backhand type, and racket type and weight). The mobile app displays all of the important data for two types of sessions: practice and match, which are set manually by the user after the session data are transferred to the user’s personal profile via Bluetooth.

**Figure 15:**
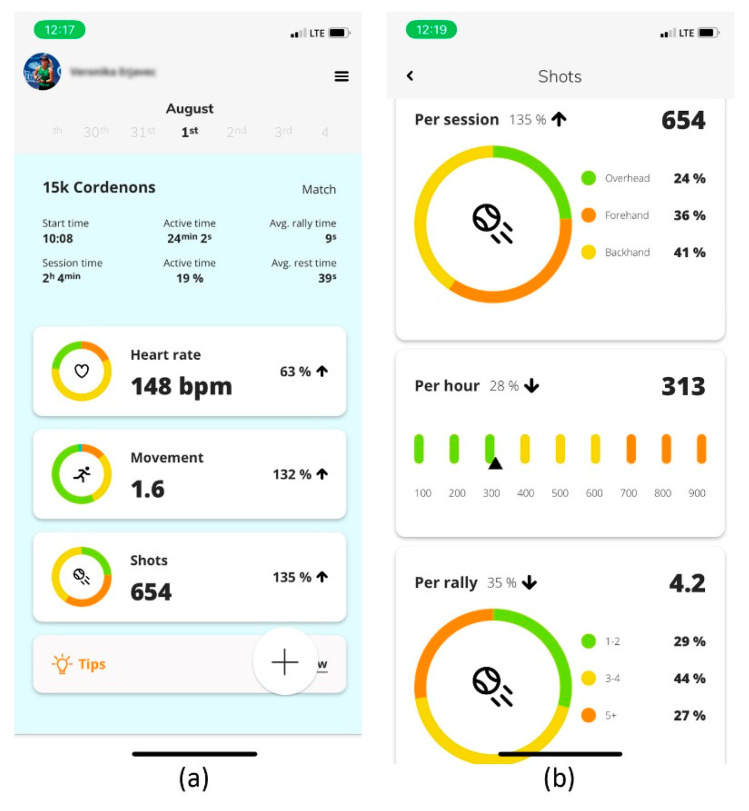
Presentation of the mobile device application graphical user interface—main window (**a**). The application shows many performance indicators clearly for intuitive user interpretation (**b**).

The first screen (Figure 15a) at the top contains personal information about the user (first and last name) and the current date, which you can expand by clicking on it to display the “Calendar”. This is followed by information about the name and the type of session (practice or match). Below that, you will find information about the session start time, duration, active time in minutes and percentages, and average rally and rest time. After the time information come three important groups of performance indicators: (1) heart rate, (2) movement, (3) shots. At the bottom of the first screen, the user can obtain quick feedback on the basic functions of the session by pressing “Tips”.

On the second screen (Figure 15b), the basic performance indicators are divided into sub-indicators that provide the user with a detailed insight into the content, workload, and quality of the performed activities. The tennis-specific group of performance indicators is divided into shots per session, shots per hour, shots per rally, shots per minute (tempo), and shot power. In terms of content, shots per session also provides extremely important information on the percentage of three types of shot: overhead, forehand, and backhand.

For a more accurate determination of workload, we developed two proprietary algorithms. The first is “cardio-load”, which defines the athlete’s total physiological load during tennis activity, while “hitting load” determines the total mechanical load on the skeletal system, especially the shoulder girdle and the dominant upper extremities. Important physiological performance indicators are the number of recoveries after 20, 60, and 120 s, which provide information on how often an athlete competed in the highest zone HR, followed by rest and recovery periods and the decreases in heart rate values. Motion data can be monitored by the user using a movement index that calculates the average speed of movement during practice or a match. The tennis-specific variables—shots, shot power, and tempo—provide information about the athlete’s technical competence and efficiency.

**Figure 16:**
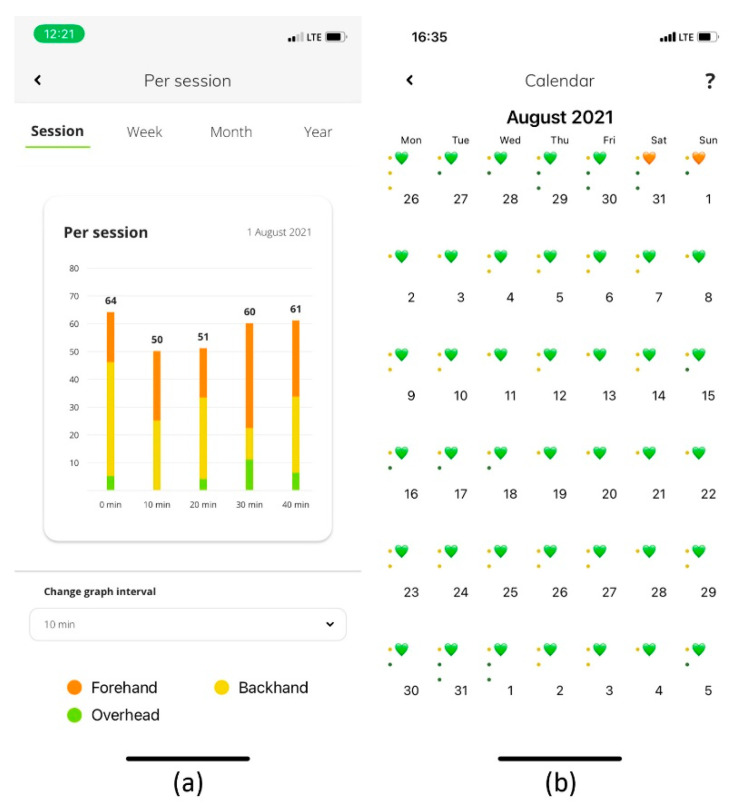
Each performance indicator can be monitored by the user from time perspectives (session, week, month, year) (**a**). Connection allows constant monitoring of a tennis player’s data by tennis or fitness and conditioning coaches, parents, or other invited staff members (**b**).

The third screen (Figure 16a) displays trends for each performance indicator during each session, week, month, and year. The trends allow the user to monitor the values of the indicators over time and to adjust the workload in specific training periods.

In addition to the performance indicators, the mobile app also contains functionalities that allow interaction between users. The connections are intended for interaction and data exchange between users. The “Summary” provides a common overview of all activities performed in different time periods. The Armbeep indicators provide the user with accurate information about the meaning and evaluation of the performance indicators. The “Calendar” displays all of the saved activities (training sessions, matches, morning HRV measurements) that the user has performed, and the Tennis Armbeep System also provides a training diary for the athlete. 

The “Calendar” is also used for one of the most important functionalities—daily planning (Figure 16b), which gives the coach and the athlete insight into the athlete’s recovery level based on the morning HRV measurements. These are listed in Table 3 and form the basis for calculating the HRV index (algorithm), which is sensitive to changes in training or match load, disease states, and, in women, the menstrual cycle. In the case of our athlete, we also found changes in the HRV index during fitness and conditioning training to improve muscular strength and power. Based on the HRV index, the calendar displays three heart colors (green, orange, red) that describe the quality of recovery and the athlete’s condition. Based on the athlete’s recovery level, the coach and athlete adjust the planned daily activities, both in terms of content and workload. Daily planning based on the measured data objectifies the degree of recovery and, together with the subjective assessment of the athlete, enables the optimal determination of the daily training load and planning of future activities. This means greater efficiency of the training activities performed, a reduction in the risk of injury and overload, and, ultimately, greater satisfaction for the athlete.

### 3.2. Limitations

To show the utility of the system, we included only one female athlete, which is a limitation, especially when applying the collected data to other age and quality categories of athlete. Another limitation is also the training and competition schedule, which was set in a very conventional way, that is, with very low training loads during the preparation period and the scheduling of the first official competitions four months after the start of training. Finally, we were limited in the conduct of the study by the number of measurement devices and athletes that were eligible for the study.

## 4. Conclusions

The presented Tennis Armbeep System is an updated version of the previous device, where the data were transferred via USB and were displayed only on a personal computer; the device did not contain such accurate data on the heart rate and movement of athletes, and the data were intended for experts and researchers. The current system is much more user-friendly, both in terms of transmission and in terms of display and interpretation of data as well as in terms of functionalities for data-based communication between users.

The system enables continuous collection of relevant data from the point of view of various performance indicators as well as analysis and planning based on personal and objective data, which experts can compare with the recommended values confirmed by research. The subjective evaluation of training load, which is still common in tennis, replaces modern data-based coaching.

An important value of the system is the possibility of daily comparison of training loads and objective evaluation of the athlete’s recovery performance. Proper dosage of workload reduces the possibility of overload and injuries significantly [39]. 

The performance indicators presented in the system have been presented and tested in several studies and cover an important part of the areas that define the workload of tennis players. For most indicators, there are reference values that allow comparison of the athlete’s personal values with the recommended values in training and competition for both genders and different age and skill groups. Users of the system are also supported by comparable standard values for individual performance indicators. Data acquisition and analysis enable the transformation of data into information and later into knowledge. Thus, the system enables a continuous learning process for the athlete and all of the experts involved.

Let us conclude by highlighting one of the objectives of the study, namely, the use of the system to monitor workload to prevent possible overuse of the athlete and, thus, the recurrence of pain. During the study, the tennis player had no pain and was able to train and compete without interruption. However, this is often a privilege rather than a constant practice in elite sports. This increases the rationality, efficiency, and systematic nature of the process of sports coaching in tennis.

## Figures and Tables

**Table 1:** Practice and match data for the observed athlete gathered in a period of six months.

		Practice		Match	
Variable ID	Description (Unit)	Mean	SD	Mean	SD
SessionTime	Session time (s)	**5805.67**	1662.91	5247.24	1520.75
ActiveTime	Active time (s)	**1961.66**	516.67	1252.65	538.12
ActiveTimePercentage	Active time (%)	**34.86**	7.68	24.03	8.68
AvgRallyTime	Average rally time (s)	**17.79**	5.11	11.81	4.33
AvgRestTime	Average rest time (s)	34.69	17.04	**37.86**	7.06
AvgHR	Average HR	129.78	9.29	**135.19**	9.63
MinHR	Min HR	79.40	8.97	**83.80**	13.07
MaxHR	Max HR	175.88	13.43	**179.35**	12.20
HighHR	Time in high-HR zone (%)	5.94	8.71	**8.49**	10.91
ModerateHR	Time in moderate-HR zone (%)	32.64	13.2	**40.8**	14.23
LowHR	Time in low-HR zone (%)	**61.37**	18.1	50.63	20.38
TotalRecoveries	Total recoveries after max or submax HR value	3.01	3.05	**67**	5.09
Recovery20Count	Number of recoveries after 20 s	3.01	3.05	**8.67**	5.09
Recovery60Count	Number of recoveries after 60 s	0.82	1.31	**1.51**	1.26
Recovery120Count	Number of recoveries after 120 s	0.00	0.00	0.00	0.00
Recovery20BPM	HR value after 20 s	3.31	3.11	**4.25**	2.38
Recovery60BPM	HR value after 60 s	15.93	10.95	**17.54**	8.43
Recovery120BPM	HR value after 120 s	0.00	0.00	0.00	0.00
CardioLoad	Cardio load index (algorithm)	55.75	43.77	**73.76**	66.02
Movement	Movement index (Valencell data)	**1.66**	0.11	1.65	0.08
Sprinting	Number of values in sprinting (%)	13.46	1.56	**13.80**	2.12
Running	Number of values in running (%)	**46.05**	7.21	39.88	8.80
Walking	Number of values in walking (%)	33.66	5.99	**43.63**	9.10
Standing	Number of values in standing (%)	**6.70**	3.53	2.69	1.99
Shots	Number of shots	**780.40**	203.69	542.86	205.67
ShotsOverhead	Number of overheads	88.16	37.59	**98.18**	39.51
POverhead	Percentage of overheads (%)	11.18	4.16	**18.20**	4.51
ShotsForehand	Number of forehands	**258.63**	86.20	166.18	87.70
Pforehand	Percentage of forehands (%)	**32.56**	5.74	29.14	7.16
ShotsBackhand	Number of backhands	**334.44**	101.43	186.80	93.84
Pbackhand	Percentage of backhands (%)	**42.32**	7.15	33.22	9.41
ShotsOther	Number of other shots	**99.17**	34.42	91.69	64.10
pOther	Percentage of other shots (%)	13.94	10.48	**19.43**	16.93
ShotsPerHour	Shots per hour	**492.54**	88.26	371.00	99.45
ShotsPerRally	Shots per rally	**7.02**	1.64	5.11	1.40
ShotsPerRallyLow	Rallies with 1–2 shots (%)	28.96	10.33	**38.96**	9.65
ShotsPerRally Moderate	Rallies with 3–4 shots (%)	**38.96**	10.11	36.37	8.80
ShotsPerRallyHigh	Rallies with 5+ shots (%)	**42.40**	13.88	24.65	12.28
ShotsPerRallyMin	Shots per rally—minimum value in session	2.00	0.00	2.00	0.00
ShotsPerRallyMax	Shots per rally—maximum value in session	**50.47**	21.53	23.12	23.05
RalliesTotal	Rallies number	**117.46**	39.45	107.67	34.93
Tempo	Shots per minute	24.02	1.86	**26.60**	2.24
TempoLow	Shots per minute (1–10 shots per minute) (%)	0.00	0.00	0.00	0.00
TempoModerate	Shots per minute (11–19 shots per minute) (%)	**16.59**	7.54	11.69	7.28
TempoHigh	Shots per minute (20+ shots per minute) (%)	83.42	7.55	**88.33**	7.28
TempoMin	Shots per minute minimum value in session	14.47	1.77	**15.36**	1.98
TempoMax	Shots per minute maximum value in session	176.76	78.73	**200.39**	59.21
ShotsPower	Shots acceleration (g)	13.40	1.79	**14.72**	2.40
ShotsPowerLow	Shots acceleration (1–10 g) (%)	32.89	12.50	**32.94**	15.01
ShotsPowerModerate	Shots acceleration (11–19 g) (%)	**55.14**	11.02	48.20	11.90
ShotsPowerHigh	Shots acceleration (20+ g) (%)	11.87	5.52	**18.69**	5.57
HittingLoad	Hitting load (algorithm) (%)	**215.18**	110.34	87.82	87.27

Note: numbers in bold represent higher average value for individual indicator for easier comparison between practice and match sessions.

**Table 2:** Values of CL (cardio load) in dependence of %*VO*_2_ and activity session duration.

	Cardio Load EPOC Value (CLe)			
%*VO*_2_	5 Min	10 Min	30 Min	60 Min
100%	0.2167	0.2167	0.2167	0.1517
90%	0.1500	0.1433	0.1517	0.0833
80%	0.1000	0.0833	0.0833	0.0683
70%	0.0667	0.0500	0.0417	0.0289
60%	0.0417	0.0250	0.0192	0.0094
50%	0.0333	0.0067	0.0067	0.0028
40%	0.0233	0.0100	0.0008	0.0006
30%	0.0167	0.0067	0.0008	0.0006

Note: Excess post-exercise oxygen consumption (EPOC) is a noninvasive method used to estimate the anaerobic energy production that occurs during exercise.

**Table 3:** Morning measurement indicators with names, description of indicators, units, and results of descriptive statistics.

Variable ID	Description	Mean	SD
MinBPM	Min HR—morning measurement	49.67	9.48
MaxBPM	Max HR—morning measurement	85.09	16.97
AvgBPM	Average HR—morning measurement	65.31	14.30
SDNN	Standard deviation of the NN (R-R) intervals	106.31	31.68
RMSSD	Reflects the integrity of vagus nerve-mediated autonomic control of the heart	122.91	42.01
pNN50	The proportion of NN50 div. by the total number of NNs	60.17	19.75
HRVScore	Variation in the time interval between heartbeats	94.68	8.80
hrv	HRV index (algorithm)	−0.05	3.30

## Data Availability

Not applicable.
